# Circulating ORM2 as a Biomarker of Metabolic Dysfunction: Evidence from the KADEM Study in Kuwaiti Adults

**DOI:** 10.3390/ijms26178326

**Published:** 2025-08-27

**Authors:** Mohamed Abu-Farha, Ahmed N. Albatineh, Bader Alawadh, Loulwa Alsalem, Irina Al-Khairi, Preethi Cherian, Fahad Al-Ajmi, Mohammad Qaddoumi, Muhammad Abdul-Ghani, Fahd Al-Mulla, Jehad Abubaker

**Affiliations:** 1 Department of Biochemistry and Molecular Biology, Dasman Diabetes Institute, Dasman 15462, Kuwait; mohamed.abufarha@dasmaninstitute.org (M.A.-F.); baderalawadh21@rcsi.ie (B.A.); irina.alkhairi@dasmaninstitute.org (I.A.-K.);; 2 Department of Translational Research, Dasman Diabetes Institute, Dasman 15462, Kuwait; 3 Department of Biostatistics and Health Data Science, College of Health, Lehigh University, Bethlehem, PA 18015, USA; 4 Pharmacology and Therapeutics Department, Faculty of Pharmacy, Kuwait University, Kuwait City 13110, Kuwait; mohammad.qaddoumi@dasmaninstitute.org; 5 Division of Diabetes, University of Texas Health Science Center, San Antonio, TX 78229, USA

**Keywords:** ORM2, diabetes, obesity, insulin resistance, ethnicity

## Abstract

Metabolic dysfunction-associated fatty liver disease (MAFLD) and type 2 diabetes mellitus (T2DM) share overlapping pathophysiological mechanisms, including insulin resistance and chronic inflammation. Recent evidence suggests that Orosomucoid-2 (ORM2), an acute-phase immunomodulatory protein, may play a role in metabolic regulation; however, its specific involvement in MAFLD remains unclear. This study examined the association between circulating ORM2 levels and the severity of hepatic steatosis, insulin resistance, and T2DM in a cohort of 449 adults. MAFLD was assessed using FibroScan^®^ with hepatic steatosis categorized by Controlled Attenuation Parameter (CAP) scores, while plasma ORM2 levels were measured via ELISA. Statistical analyses using Spearman correlation and multiple logistic regression revealed that elevated ORM2 levels were significantly correlated with greater hepatic steatosis, insulin resistance, triglycerides, ALT, and hip circumference (*p* < 0.001). Individuals with severe steatosis (CAP > 290 dB/m) had markedly higher ORM2 levels (312.3 ng/mL) compared to those with normal CAP scores (210.4 ng/mL; *p* < 0.001). ORM2 was identified as an independent predictor of steatosis severity and after adjusting for several metabolic variables (AOR = 1.005; 95% CI: 1.002–1.007). ROC analysis incorporating ORM2 and metabolic variables demonstrated strong predictive capability for MAFLD (AUC = 0.864, 95% CI: 0.825–0.902). These findings support ORM2 as a promising non-invasive diagnosis for MAFLD, involving only blood sampling without direct invasion of the liver and associated metabolic dysfunction.

## 1. Introduction

MAFLD is a complex condition that includes a spectrum of liver pathologies, ranging from simple hepatic steatosis to metabolic dysfunction-associated steatohepatitis (MASH), which may progress to liver cirrhosis and hepatocellular carcinoma (HCC) [1]. The primary pathological features of MAFLD include hepatic steatosis, inflammation, and fibrosis, which have been the focus of various therapeutic interventions, with promising results in targeting these components in a significant proportion of affected individuals [1]. Given the rising prevalence of metabolic disorders, understanding the molecular mechanisms contributing to MAFLD pathogenesis is crucial for developing effective diagnostic and therapeutic strategies.

MAFLD and T2DM are closely intertwined, with each condition exacerbating the other. Recent studies have shown that the global prevalence of T2DM among individuals with MAFLD is approximately 28.1% [2]. This significant overlap emphasizes the bidirectional relationship between these diseases, where MAFLD not only increases the risk of developing T2DM but also worsens its prognosis [2]. The shared pathophysiological mechanisms, including insulin resistance, chronic inflammation, and metabolic dysregulation, contribute to the progression of both conditions [2]. Recognizing this association is crucial for developing early intervention and management strategies to reduce the burden of these interrelated diseases.

ORM, also known as alpha-1-acid glycoprotein (AGP), is a major acute-phase plasma protein with significant immunomodulatory functions. It exists in two primary isoforms, ORM1 and ORM2, which differ by 22 amino acids [3]. Fu et al. (2025) reported that in a cohort of 2270 adult women from the NHANES database, AGP showed a strong positive association with NAFLD and liver fibrosis, with an inverted U-shaped relationship to CAP scores, suggesting that AGP levels may have a non-linear link with hepatic steatosis severity [4]. ORM2 is implicated in both pro-inflammatory and anti-inflammatory pathways, with a potential role in immune modulation [5,6]. Additionally, ORM2 exerts inhibitory effects on platelet aggregation, lymphocyte proliferation, and neutrophil chemotaxis through mechanisms that remain to be fully explained [7]. Emerging evidence suggests that ORM2 may also play a role in neuroinflammation, as demonstrated in a study where it was found to exhibit anti-inflammatory properties in the central nervous system [8]. However, the relationship between ORM2 and MAFLD remains unclear. Recent findings suggest that ORM2 functions as a hepatokine induced by bile acids, playing a critical role in metabolic regulation [9,10]. Bile acids are known to improve glucose metabolism and insulin sensitivity [9,10]. The study by Lee et al. (2024) identified ORM2 as a key mediator of bile acid-induced metabolic benefits, particularly through its effects on adipose tissue inflammation [10]. ORM2 was found to be essential for bile acid-induced metabolic improvements in obesity [10].

This study aims to investigate the association between ORM2, MAFLD, T2DM, and insulin resistance in individuals from the Kuwait Adult Diabetes and Epidemiological Multidisciplinary (KADEM) program. In addition, this study aims to assess the use of ORM2 as a potential non-invasive diagnosis for MAFLD, involving only blood sampling without direct invasion of the liver. This explores the impact of ORM2 on key metabolic factors, including insulin levels, glucose metabolism, and adiposity, to determine its potential as a biomarker for MAFLD severity.

## 2. Results

**Figure 1:**
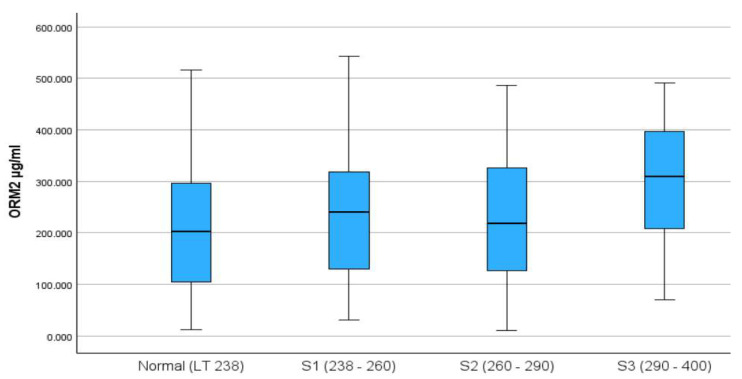
Distribution of the ORM2 (ng/mL) biomarker across levels of the CAP (dB/m) score for the study sample.

The final analysis included 449 participants, of whom 52% were males, with a median (IQR) age of 49 (15) years. The distribution of ORM2 by CAP score categories is presented in Figure 1, with the S3 category having the highest median ORM2 levels. Table 1 presents the demographic and clinical characteristics of participants by CAP scores, where a score of ≤290 is considered a normal score and a score of >290 is regarded as high. Upon dichotomization of the CAP score to normal (77.3%) and high (22.3%) categories, the descriptive analysis indicated a marginal association between gender and CAP score, with 26.1% of males having high CAP scores compared to 18.3% among females. The results indicated that in the high CAP score group, the medians for hip circumference, HDL, TG, ALT, AST, insulin, glucose, and ORM2 were significantly higher compared to the normal CAP score group; however, no significant differences in medians were noticed for age, TC, and LDL covariates, with details presented in Table 1.

To better understand the strength of the relationship between the CAP score and other covariates, the Spearman correlation coefficient indicated a significant positive correlation between the CAP score and all covariates, except for HDL, in which the correlation was significant but negative. Furthermore, the point-biserial correlation indicated that males tend to have significantly higher ORM2 compared to females, with details presented in Table 2.

To model the association between ORM2 as the primary exposure and the CAP score as a binary outcome, results from the multiple logistic regression are presented in Table 3. Univariate logistic regression analysis indicated that ORM2, glucose, insulin, ALT, and hip circumference are independently and significantly associated with high CAP scores, as presented in Table 3. In the adjusted analysis, ORM2 (AOR = 1.005, 95% CI: 1.002, 1.007), ALT (AOR = 1.044, 95% CI: 1.028, 1.062), insulin (AOR = 1.048, 95% CI: 1.023, 1.073), glucose (AOR = 1.213, 95% CI: 1.022, 1.440), and hip circumference (AOR = 1.072, 95% CI: 1.042, 1.104) are significantly associated with an increased odds of having high CAP score. For example, with a one-unit (ten-unit) increase in ORM2, there is a 0.5% (5.1%) increase in the odds of having a high CAP score after controlling for all other covariates included in the model. Similarly, with a one-unit increase in hip circumference, ALT, insulin, and glucose, there is a 7.2%, 4.4%, 4.8%, and 21.3% increase in the odds of having a high CAP score when controlling for the effects of all other covariates in the model, with details presented in Table 3.

**Figure 2:**
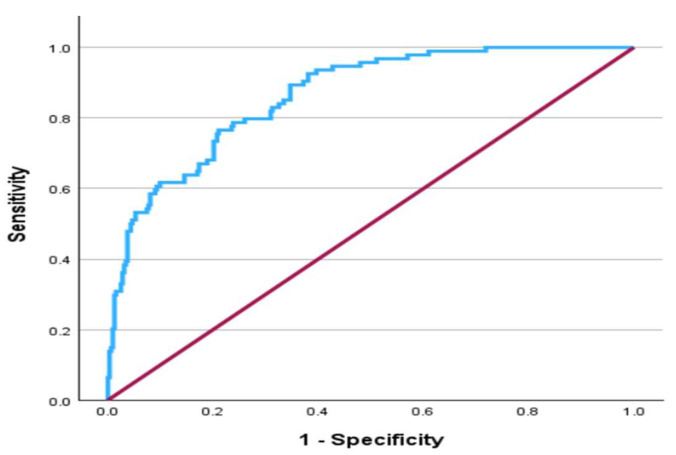
The area under the Receiver Operating Characteristics Curve represents the predictive ability of the model presented in Table 3.

**Figure 3:**
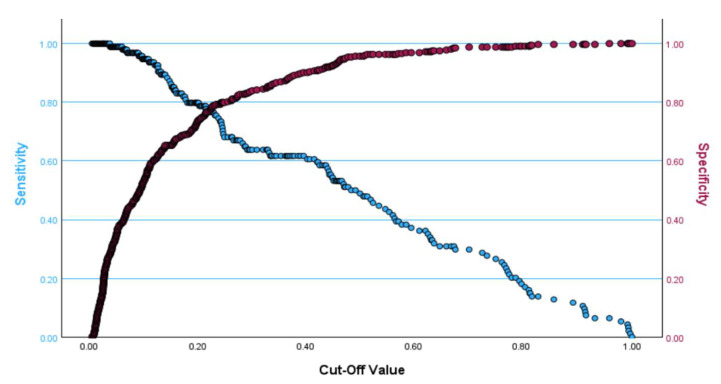
Optimal cut-off point at which both sensitivity and specificity are maximized for the model presented in Table 3 (Sensitivity = 0.766; Specificity = 0.789).

Finally, the area under the curve (AUC) of the Receiver Operating Characteristic (ROC) was estimated to assess the predictive ability of the multiple logistic regression model in identifying cases and non-cases of high CAP scores and to show the effect of each covariate in a stepwise manner. The AUC for the final model was 0.864 (95% CI: 0.825, 0.902), which is a very good discriminating ability and is displayed in Figure 2. The effect of the covariates on the AUC in a stepwise manner is presented in Table 4. If the cut-off value produced by the Youden index was used, the cut-off values of OMR2 for males and females were estimated as 275.92 and 285.58, respectively. The optimal cut-off point at which both sensitivity and specificity are maximized for the final model in Table 3 is approximately 0.20 and is presented in Figure 3, where the sensitivity is equal to 0.766 and the specificity is equal to 0.789. It is worth mentioning that the multiple logistic regression model is significant, according to the omnibus test (chi-square = 142.3, DF = 7, *p* < 0.001) and fits the data well according to the Hosmer–Lemeshow test (Chi-square = 9.92, DF = 8, *p* = 0.271).

## 3. Discussion

The principal finding of this study was that elevated ORM2 levels significantly correlated with increased severity of metabolic dysfunction, specifically hepatic steatosis, insulin resistance, and levels of TG and ALT. Individuals with high CAP scores (>290 dB/m) had notably higher median ORM2 concentrations compared to those with normal scores (312.3 vs. 210.4 µg/mL, *p* < 0.001). Moreover, logistic regression analyses underscored ORM2 as an independent predictor of severe hepatic steatosis, with each unit increase in ORM2 associated with 0.5% greater odds of severe steatosis (AOR = 1.005; 95% CI: 1.002–1.007, *p* < 0.001). While ORM2 shows a statistically significant independent association with MAFLD, the effect size on its own is modest. Its greatest predictive value is observed when combined with other clinical and metabolic markers in a multivariate model.

Consistent with prior studies, our findings support the relationship between elevated ORM2 and metabolic dysregulation. Previous research indicates that ORM2 plays a significant role in lipid metabolism and inflammation, potentially through AMP-activated protein kinase signaling pathways, which are critical in metabolic and inflammatory regulation [11,12]. Our results align with earlier observations showing that markers of metabolic dysfunction, including triglycerides, insulin, and liver enzymes, correlate positively with hepatic steatosis and negatively with HDL cholesterol [13,14,15,16,17,18]. Thus, this study contributes additional evidence confirming the involvement of ORM2 in metabolic pathogenesis, enhancing the current understanding of biomarkers for metabolic diseases.

The biological role of ORM2 in hepatic lipid metabolism and insulin signaling remains to be fully elucidated. However, prior work has shown that ORM2 is regulated by bile acids and may mediate their insulin-sensitizing effects via adipose tissue inflammation pathways [9]. Given these mechanistic links, elevated ORM2 in MAFLD could represent a compensatory response to hepatic lipid overload and inflammation. Alternatively, ORM2 may actively participate in disease progression through modulation of immune or metabolic signaling cascades.

Importantly, multivariable logistic regression analysis demonstrated that ORM2 remained a statistically significant independent predictor of high CAP scores (AOR = 1.005, 95% CI: 1.002–1.007, *p* < 0.001), even after adjusting for age, gender, and other metabolic risk factors. This reinforces its potential role as a non-invasive diagnosis for MAFLD, involving only blood sampling without direct invasion of the liver, particularly in high-risk populations such as individuals with obesity or insulin resistance. Furthermore, the model incorporating ORM2 along with age, gender, hip circumference, ALT, insulin, and glucose achieved a high AUC of 0.864 (95% CI: 0.825–0.902), indicating excellent discriminative power in identifying individuals with high hepatic fat content. Furthermore, gender-specific analysis yielded different ORM2 cut-off values for males and females (275.92 ng/mL and 285.58 ng/mL, respectively), which may reflect underlying sex differences in fat distribution, inflammatory response, or ORM2 expression patterns. Future research should explore whether sex-specific thresholds improve clinical accuracy when applying ORM2 as a diagnostic tool in broader populations. Fu et al. (2025) conducted an interesting study that involved data for a cohort of 2270 adult women extracted from the NHANES database. Their results indicated that alpha-1-acid glycoprotein (AGP), which is a major acute-phase plasma protein with significant immunomodulatory functions, exists in two primary isoforms, ORM1 and ORM2, and has a strong positive association with NAFLD and liver fibrosis, with an inverted U-shaped relationship to CAP scores. This suggests that AGP levels may have a non-linear link with hepatic steatosis severity. On the other hand, the current study revealed that elevated ORM2 levels were significantly correlated with greater hepatic steatosis, insulin resistance, triglycerides, ALT, and hip circumference. Furthermore, the current study showed that individuals with severe steatosis (CAP > 290) had markedly higher ORM2 levels compared to those with normal CAP scores. However, it is essential to note that the current study focuses on ORM2, with participants comprising a mixture of males and females from different ethnicities (Arab vs. non-Arab), whereas the study by Fu et al. (2025) focused exclusively on adult women.

Several limitations should be acknowledged. Firstly, the cross-sectional nature of this study does not allow for the establishment of a causal relationship between ORM2 elevation and metabolic dysfunction. Longitudinal studies are needed to clarify whether elevated ORM2 precedes disease onset or results from metabolic disorders. Secondly, this study’s generalizability may be limited due to its focus solely on Kuwaiti adults, and the observed associations might differ in other ethnic or cultural contexts. Future studies involving diverse populations are essential to validate our findings comprehensively. Lastly, despite demonstrating clear associations, the precise molecular mechanisms through which ORM2 influences metabolic pathways remain uncertain, necessitating detailed mechanistic research.

Future studies should adopt longitudinal designs to elaborate on the causal relationship between ORM2 levels and metabolic disease progression. Investigating the predictive value of ORM2 through prospective studies could enhance its clinical utility. Additionally, experimental research should focus on uncovering the molecular mechanisms underpinning ORM2′s role in metabolic regulation, potentially identifying novel therapeutic targets. Lastly, validating these findings across diverse ethnic groups could establish universal applicability or identify necessary adjustments in ORM2 thresholds based on specific population characteristics.

## 4. Materials and Methods

### 4.1. Study Design and Population

The KADEM program is a cross-sectional study conducted at the Dasman Diabetes Institute, which was approved by the DDI ethical committee (Study Number RA-2019-030) and registered on clinicaltrials.gov (NCT06115876, accessed on 23 May 2022). This study was conducted in accordance with the ethical framework of the Helsinki Declaration, in which random sampling of the Kuwaiti population with proportional representation from each of the seven governorates was conducted for participant recruitment. A list of Kuwaiti residents, complete with their unique identification codes, was provided by the National Public Authority of Civil Information. A stratified random sampling technique was employed to select survey participants from this resident list. The survey design was adapted from the WHO STEPwise approach to surveillance (STEPS) methodology. All participants signed a consent form before they participated in this study. This study includes 449 participants assessed for MAFLD using FibroScan^®^ (Echosens, Paris, France). Individuals suffering from any infection and those aged younger than 18 or older than 65 were excluded from this study. MAFLD stages were classified based on hepatic steatosis severity according to the CAP score into four groups: normal (<238 dB/m), S1 (238–260 dB/m), S2 (261–290 dB/m), and S3 (>290 dB/m). Participants underwent routine clinical blood tests, and measurements of Body Mass Index (BMI) and hip circumference were recorded. To assess clinical variables, participants were asked to fast for at least 10 h before blood samples were collected to measure lipid and glycemic profiles, including triglycerides (TG) hemoglobin A1c (HbA1c), insulin, fasting plasma glucose (FPG), total cholesterol (TC), high-density lipoprotein (HDL-C), and low-density lipoprotein (LDL-C). Glucose and lipid profiles were assessed using the Siemens Dimension RXL chemistry analyzer (Diamond Diagnostics, Holliston, MA, USA), whereas HbA1c levels were determined using the VariantTM device (BioRad, Hercules, CA, USA). All laboratory assessments were conducted by certified technicians at the clinical laboratories of DDI, following approved methods and quality standards established by the Ministry of Health. Insulin levels were quantified using the Access Ultrasensitive Insulin Assay (Beckman Coulter, Brea, CA, USA), with both intra- and inter-assay coefficients of variation not exceeding 6%. Insulin resistance was calculated using the Homeostatic Model Assessment for Insulin Resistance (HOMA-IR) formula: (FBG in mmol/L) × (fasting insulin in mU/L)/22.5. Multiple logistic regression was used to evaluate the discriminative ability of ORM2 and other covariates in predicting high CAP scores, with the area under the receiver operator characteristic curve (AUC), sensitivity, and specificity reported.

### 4.2. Sample Preparation

Blood samples were collected in EDTA tubes and centrifuged at 400× *g* for 10 min at room temperature to separate the plasma. The plasma samples were stored at −80 °C until used. For the ELISA assay, the required plasma samples were thawed at room temperature, then centrifuged at 10,000× *g* for 5 min to remove any particulates or precipitates. The plasma samples were aliquoted into the appropriate plate layout.

### 4.3. Measurement of Circulating Levels of ORM2 by ELISA

The human ORM2 levels in plasma were determined using the Human ORM2 (Alpha-1-acid glycoprotein 2) ELISA Kit (Cat. No. orb563348) from Biorbyt Ltd. (Cambridge, UK) The plasma samples were diluted 1:50,000 using the 1× sample buffer provided in the kit. The ELISA was performed according to the manufacturer’s instructions. Briefly, standards and diluted samples were pipetted into the pre-coated microplate wells and incubated for 90 min at 37 °C. After incubation, the wells were washed extensively to remove unbound components. A biotinylated monoclonal antibody specific to human ORM2 was then added to the wells. Following the removal of excess antibody, streptavidin–horseradish peroxidase (STREP-HRP) conjugate was added. After a final washing step, the peroxidase activity was quantified by adding the substrate solution. The reaction was stopped by adding the stop solution, and the color intensity was measured at 450 nm using an ELISA plate reader (Synergy HTX from BioTek Instruments, Inc., Winooski, VT, USA). The color intensity is directly proportional to the concentration of human ORM2 in the samples. The concentration of ORM2 in the samples was calculated by plotting the sample absorbance values against a standard curve. The intra-assay and inter-assay coefficients of variation (CV%) were both less than 10%.

### 4.4. Statistical Analysis

The statistical analysis was conducted using the Statistical Package for Social Sciences (SPSS) (IBM Corp, Version 29) and R statistical software (R Core Team). The data was imported, checked for abnormalities, and then coded for descriptive purposes. To describe the center and variability, continuous variables were reported as mean and SD if normality was satisfied; otherwise, the median and Interquartile Range (IQR) were reported. The two-sample *t*-test was used to test the equality of means if the normality of the two groups was satisfied; otherwise, the Mann–Whitney U test was implemented to compare medians. To test for association between two categorical variables, the Pearson chi-square test of independence was implemented if the expected cell counts for ≥80% of the cells were more than 5; otherwise, the Fisher exact test was implemented. The Spearman correlation coefficient was implemented due to the presence of outliers to measure the strength of the linear relationship between two continuous variables.

The main outcome was the CAP score, which, according to the steatosis grade has the following categorization: normal (<238 dB/m), S1 (238–259 dB/m), S2 (260–290 dB/m), and S3 (>290 dB/m). Since our goal was to estimate the odds of patients with higher CAP scores using an array of covariates, a two-level outcome (normal ≤ 290 dB/m, high > 290 dB/m) was derived and implemented in the analysis.

A multiple logistic regression model was implemented to model the association between the CAP score (binary outcome: 0 = normal, 1 = high) and several covariates, and the odds ratios with their corresponding 95% confidence intervals (CI) were reported. The multiple logistic regression model was assessed for significance using an omnibus test based on the chi-square test statistic, and the model fit was assessed using the Hosmer–Lemeshow test. To make sure that the interpretations of the estimates for the continuous covariates are sensible, the linearity assumption between each of the continuous covariates and the log of the odds was checked.

The AUC was reported to assess the multiple logistic regression model’s predictive ability as a discriminating tool. Furthermore, the cut-off value for the logistic regression model’s main exposure was reported based on the optimal cut-off value produced by the Youden index. All tests were two-tailed, and a significance level was set to 5%.

## 5. Conclusions

In conclusion, this study establishes circulating ORM2 as a significant and independent predictor of hepatic steatosis severity and metabolic dysfunction. With strong performance in ROC analysis and robust associations with insulin resistance and liver enzyme levels, ORM2 holds promise as a non-invasive diagnosis for MAFLD, involving only blood sampling without direct invasion of the liver. Future longitudinal studies are needed to assess ORM2′s prognostic value and to elucidate its mechanistic roles in metabolic disease pathways.

## Figures and Tables

**Table 1:** Descriptive analysis of the distribution of CAP (dB/m) score categories with some biomarkers, including ORM2 (^c^ N = 449).

Covariate *	CAP Score Categories		PV
	Normal (≤290 dB/m)	High (>290 dB/m)	
Gender, n (%) Male Female	170 (73.9) 174 (81.7)	60 (26.1) 39 (18.3)	0.053 ^a^
Age (years)	48.0 (16)	50 (10)	0.194 ^b^
Hip Circumference (cm)	105 (15)	111 (17)	<0.001 ^b^
TC mmol/L	5.1 (1.51)	5.3 (1.15)	0.181 ^b^
LDL-C mmol/L	3.2 (1.3)	3.3 (1.15)	0.113 ^b^
HDL-C mmol/L	1.49 (0.45)	1.32 (0.33)	<0.001 ^b^
TG mmol/L	0.90 (0.62)	1.31 (0.95)	<0.001 ^b^
ALT U/L	27 (14)	37 (27)	<0.001 ^b^
AST U/L	20 (7)	21 (14)	0.007 ^b^
Insulin mU/L	10.2 (10.6)	19.8 (19.1)	<0.001 ^b^
Glucose mmol/L	5.1 (1.0)	5.8 (1.7)	<0.001 ^b^
ORM2 (µg/mL)	210.4 (191.1)	312.3 (185.3)	<0.001 ^b^

^a^ Calculated using the chi-square test of independence. ^b^ Calculated using the Mann–Whitney U test due to non-normality of at least one group. ^c^ Counts may not add up to N due to a few missing values. Biomarkers: TC: total cholesterol; LDL-C: low-density lipoprotein cholesterol; HDL-C: high-density lipoprotein cholesterol; TG: triglycerides; ALT: alanine transaminase; and AST: aspartate transaminase. * Except for gender, values reported are median (IQR).

**Table 2:** Spearman correlations between ORM2 (µg/mL), CAP score (dB/m), and other biomarkers (N = 449).

Covariate	Correlation (PV)
Gender	0.149 (0.002)
Age (years)	0.249 (<0.001)
Hip Circumference (cm)	0.342 (<0.001)
TC mmol/L	0.120 (0.012)
LDL-C mmol/L	0.132 (0.006)
TG mmol/L	0.455 (<0.001)
HDL-C mmol/L	−0.308 (<0.001)
ALT U/L	0.352 (<0.001)
AST U/L	0.157 (0.001)
Insulin mU/L	0.478 (<0.001)
Glucose mmol/L	0.340 (<0.001)
CAP (dB/m)	0.288 (<0.001)

Correlation (PV): probability value. Biomarkers: TC: total cholesterol; LDL-C: low-density lipoprotein cholesterol; HDL-C: high-density lipoprotein cholesterol; TG: triglycerides; ALT: alanine transaminase; and AST: aspartate transaminase. CAP: Controlled Attenuation Parameter is a measurement used in liver health assessments, specifically with FibroScan testing.

**Table 3:** Association between the CAP (dB/m) score and ORM2 (µg/mL), along with anthropometric covariates using multiple logistic regression analysis (N = 449).

Covariate	Univariable Analysis		Adjusted Analysis	
	OR (95% CI)	PV	AOR (95% CI)	PV
Hip circumference (cm)	1.068 (1.046, 1.090)	<0.001	1.072 (1.042, 1.104)	<0.001
ALT U/L	1.040 (1.026, 1.054)	<0.001	1.044 (1.028, 1.062)	<0.001
Insulin mU/L	1.080 (1.057, 1.103)	<0.001	1.048 (1.023, 1.073)	<0.001
Glucose mmol/L	1.279 (1.130, 1.447)	<0.001	1.213 (1.022, 1.440)	0.027
ORM2 (µg/mL)	1.006 (1.004, 1.008)	<0.001	1.005 (1.002, 1.007)	<0.001

OR: odds ratio, AOR: adjusted odds ratio, PV: probability value. ORs were adjusted for “age” and “gender”.

**Table 4:** Stepwise analysis of the area under the ROC curve for predicting the odds of high CAP (dB/m) score using ORM2 (µg/mL) and anthropometric covariates (N = 449).

Model/Covariates Included	* AUC (95% CI)	Optimal Cut-Off Value
ORM2	0.708 (0.651, 0.765)	Male = 275.92 Female = 285.58
ORM2 + Gender + Age	0.728 (0.676, 0.781)	
ORM2 + Gender + Age + Hip Circumference	0.782 (0.732, 0.831)	
ORM2 + Gender + Age + Hip Circumference + ALT	0.835 (0.790, 0.881)	
ORM2 + Gender + Age + Hip Circumference + ALT + Insulin + Glucose	0.864 (0.825, 0.902)	

* AUC: area under the curve. Optimal Cut-Off values are calculated based on the Youden index.

## Data Availability

The datasets used and/or analyzed during this study are available from the corresponding author upon reasonable request.

## Abbreviations

The following abbreviations are used in this manuscript.

MAFLD	Metabolic dysfunction-associated fatty liver disease
CAP	Controlled Attenuation Parameter
ORM2	Orosomucoid-2
T2DM	Type 2 diabetes mellitus
AUC	The area under the receiver operator characteristic curve
